# Periorbital Necrotising Fasciitis after Minor Skin Trauma

**DOI:** 10.1155/2014/723408

**Published:** 2014-09-21

**Authors:** Ceren Günel, Aylin Eryılmaz, Yeşim Başal, Ali Toka

**Affiliations:** Department of Otorhinolaryngology-Head and Neck Surgery, Medical Faculty, Adnan Menderes University, KBB AD, Aytepe Mevkii, 09100 Aydın, Turkey

## Abstract

Necrotizing fasciitis (NF) is a fatal and rare disease, mainly located in extremity and body. Due to the good blood supply, the occurrence of this infective disease of skin and subcutaneous tissue/fascia is much rarer in the head and neck region. In this study, we represent periorbital necrotizing fasciitis case in a patient with normal immune system. The patient applied the emergency clinic with the complaints of swelling and redness on the left eye. It was found out that a skin incision occurred at 2 cm below the left eye with razor blade 2 days ago. After taking swab culture sample, patient was started on parenteral Vancomycin + Ampicillin-Sulbactam treatment. It was observed that necrosis spread within hours and an emergent deep surgical debridement was performed. Following the debridement, it was observed that periorbital edema began to regress prominently on the 1st day of the treatment. Treatment was carried on with daily wound care and parenteral antibiotherapy. The patient was discharged from the hospital with slightly cosmetic defect.

## 1. Introduction

Necrotizing fasciitis (NF) is a fatal and rare disease, mainly located in extremity and body. Due to the good blood supply, the occurrence of this infective disease of skin and subcutaneous tissue/fascia is much rarer in the head and neck region. Mortality rate of periorbital necrotizing fasciitis is reported between 10% and 14.2% on average [1–3]. Mortality generally develops due to systemic complications such as septicemia and multiple organ failures [4, 5].

Periorbital NF frequently is accompanied by trauma, immunosuppression, alcoholism, and polymyositis [3]. In this study we represent a case which started with a wound site infection after a minor skin trauma and rapidly progressed to necrotizing fasciitis.

## 2. Case

**Figure 1:**
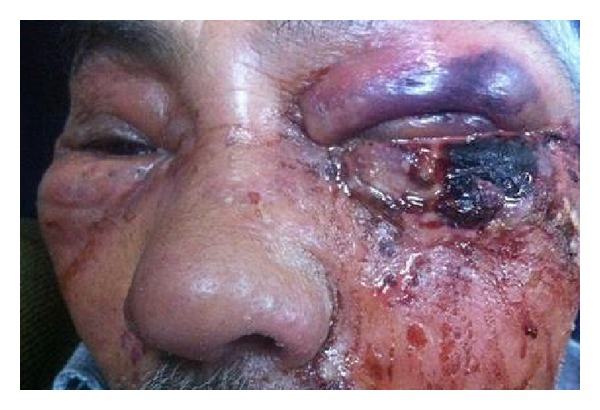
Anterior view of the patient at the emergency clinic.

**Figure 2:**
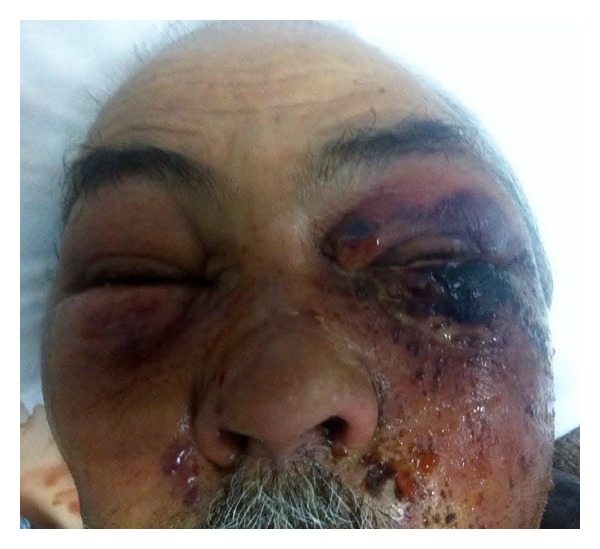
The necrosis spread within hours.

A 75-year-old male patient admitted to emergency service because of swelling and redness on the left eye and wound discharge under the eye. It was found out that a skin incision occurred at 2 cm below the left eye with razor blade 2 days ago. Redness, swelling, and efflux developed in this incision region within 2 days progressively. Patient specified that the periorbital region crusted over. In medical history of the patient, no additional disease or a condition related with immunosuppression was observed except having bypass due to coronary artery disease. In the physical examination, left periorbital area was hyperemic and edematous and temperature was increased in this area. Under the left eyelid, an irregular 2 cm skin lesion with discharge and necrosis was observed. There was a widespread edema at the left side of the face. There was a minimal periorbital edema at the right site (Figure 1). High fever was present as a pathological vital finding. Patient's otorhinolaryngological examination was normal otherwise. A neutrophil dominant leukocytosis (15.000/mkrl) was observed in CBC. Sedimentation, 78 mm, and C-reactive protein (CRP), 393 mg/L, were detected. Biochemical parameters were normal. In the ophthalmologic examination, loss of sight was present in the left eye (0/1 snellen). In the anterior segment examination chemosis, periorbital edema, and echimosis were seen. Chorioretinal atrophy was detected in the dilated fungus assessment. In the orbital magnetic resonance examination, rise in the density of periorbital adipose tissue was observed. After taking swab culture sample, patient was started on parenteral Vancomycin + Ampicillin-sulbactam treatment. It was observed that necrosis spread within hours (Figure 2). Since the clinical situation continued to progress, an emergent deep surgical debridement was performed. Pathology specimens were taken. Wound site care was made by the application of pomades with topical antibiotics and topical rifampicin. Following the debridement, it was observed that periorbital edema began to regress prominently on the 1st day of the treatment. Treatment was carried on with daily wound care and parenteral antibiotherapy. Wound site culture resulted as positive for Streptococcus pyogenes on the 3rd day of the treatment. Patient's antibiotherapy was rearranged as stopping the vancomycin therapy and continuing 1.5 g parenteral ampicillin sulbactam therapy (4 times/day) based on the consultation of department of infectious disease. In the 3rd week, wound healing speeded up and debrided areas got epithelized. The patient was discharged from the hospital with slightly cosmetic defect. In the 1st month, periorbital fasciitis completely disappeared. Unfortunately, the patient failed to attend for follow-up.

## 3. Discussion

NF was first described in 1952 by Wilson. Necrotizing fasciitis is a fatal disease and is rarely seen in the head and neck region. Incidence of periorbital NF is low due to the rich blood supply of this area [6]. Mortality due to the periorbital necrotizing fasciitis is reported between 10% and 14.2% on average [1–3]. This is far less compared to NF in the other regions of the body, which can vary from 20% to 35% [5]. Mortality generally develops due to systemic complications such as septicemia and multiple organ failures [4, 5].

Although, NF frequently generally develops secondary to penetrating trauma or surgery, it may also appear in case of immunosuppression, alcoholism, malignancy, and trauma [3]. Such predisposing factors should be well examined by clinicians. Periorbital NF was also reported after trauma [7] and surgical operations such as dacryocystorhinostomy [4] and blepharoplasty [8]. Although there were no predisposing factors like diabetes mellitus and malignancy in our case, a recent skin trauma was present.

NF is clinically initiated by periorbital edema and redness. This appearance frequently resembles preceptal cellulitis and erysipelas. However, blackish skin color change and crust form should be a warning for necrotizing fasciitis. Thin skin can provide an early diagnosis of the disease. Progressive vascular thrombosis rapidly causes necrosis of subcutaneous tissue [5]. The subcutaneous necrosis is usually more extensive than that suggested by the changes in the overlying skin; however muscular layer is generally retained [7]. In the progressive state, crepitation can be determined with palpation. Air density in the soft tissue can be determined by X-ray imaging.

NF is investigated in 2 subgroups according to microbiological culture. Type I is originated from polymicrobial pathogens and frequently seen at immunosuppressive individuals. Type II is frequently originating from single agent such as Streptococcus pyogenes or Staphylococcus aureus. The most frequent observed agents are Streptococcus pyogenes or Staphylococcus aureus [6]. Bacterial neurotoxins are responsible for the tissue damage. Moreover, it can cause complications such as glomerulonephritis and endocarditis [6]. After culture sampling, empiric treatment can be initiated with parenteral broad-spectrum antibiotics. According to the culture results, a rearrangement in antibiotherapy according to the pathogenic bacteria can be made. Standard antimicrobial therapy should include beta-lactam antibiotic such as penicillin or cephalosporin. Benzyl penicillin is effective against Streptococcus pyogenes. Clindamycin can be recommended since it decreases the streptococcal toxins and enzymes in the subinhibitor concentration [9]. In this case, broad spectrum antibiotherapy was initiated after culture sampling and ampicillin sulbactam treatment was applied after Streptococcus pyogenes reproduction in the culture.

High dose antibiotic combinations initiated with early diagnosis and tissue debridement will help to decrease the mortality. Mild cases can also respond to antibiotic treatment only [6, 10]. Since thrombosis developed in the blood vessel, antibiotics may not be effective in the infected region. Therefore, antibiotic therapy must be combined with an appropriate surgical debridement. All necrotic tissues must be debrided until reaching the live hemorrhagic tissue in the surgical debridement. During the debridement, underlying muscles and eyelids must be protected in order to prevent ectropion and keratitis. In case of a slow response to the therapy, repeated debridement surgeries can be considered [3, 11]. After taking the acute phase under control, a reconstructive surgery can be planned for a further date.

In conclusion, periorbital necrotizing fasciitis is a rare disease and difficult to diagnose. Darkening at the lesion area can be a clinical warning sign. Even though a follow-up with antibiotherapy can be preferred in mild-moderate necrotizing fasciitis, a late surgical debridement will lead to increase in mortality and morbidity since the progression of the necrosis is very rapid. Effective clinical diagnosis and treatment is the most important factor in order to decrease the mortality and morbidity.
